# Leaf Nutritional Content, Tree Richness, and Season Shape the Caterpillar Functional Trait Composition Hosted by Trees

**DOI:** 10.3390/insects13121100

**Published:** 2022-11-29

**Authors:** Perttu Anttonen, Yi Li, Douglas Chesters, Andréa Davrinche, Sylvia Haider, Helge Bruelheide, Jing-Ting Chen, Ming-Qiang Wang, Ke-Ping Ma, Chao-Dong Zhu, Andreas Schuldt

**Affiliations:** 1 Institute of Biology/Geobotany and Botanical Garden, Martin Luther University Halle-Wittenberg, 06108 Halle, Germany; 2 German Centre for Integrative Biodiversity Research (iDiv) Halle-Jena-Leipzig, 04103 Leipzig, Germany; 3 State Key Laboratory of Vegetation and Environmental Change, Institute of Botany, Chinese Academy of Sciences, Beijing 100093, China; 4 Key Laboratory of Zoological Systematics and Evolution, Institute of Zoology, Chinese Academy of Sciences, Beijing 100101, China; 5 International College, University of Chinese Academy of Sciences, Beijing 100049, China; 6 College of Biological Sciences, University of Chinese Academy of Sciences, Beijing 100049, China; 7 State Key Laboratory of Integrated Pest Management, Institute of Zoology, Chinese Academy of Sciences, Beijing 100101, China; 8 Department of Forest Nature Conservation, Georg-August-University Göttingen, 37077 Göttingen, Germany

**Keywords:** body weight, carbon, defense, generalist, leaf traits, Lepidoptera, magnesium, nitrogen, plant richness, specialist

## Abstract

**Simple Summary:**

The nutritional content of food plants can, to a large extent, determine the physical attributes of herbivorous insects, from growth rates to the need for defenses against predators. In forests, tree species richness may influence these plant-mediated effects through increasing variation in the nutritional quality that herbivorous insects encounter. Seasonal progression can also shape the plant–herbivore relationship, with lowered leaf quality in later seasons. It is expected that specialist herbivores fare better than generalists in poorer nutritional-quality host plants, whereas generalists can benefit from dietary mixing in more variable neighborhoods. However, a clear understanding of how these factors interact to influence the diversity and functionality across multiple traits of herbivorous insect communities is lacking. In this study, we found support for the expectation of higher generalism of caterpillars in high-nutrition content trees, which also promoted higher abundance but lowered caterpillar species richness and smaller and less defended individuals. Increasing tree richness led to higher caterpillar species sharing between tree species, decreased trait variation, and increased caterpillar species richness per tree species. Our findings shed light on how leaf traits and changes in tree richness interact to influence the trait composition of key herbivores through fine-scale habitat partitioning in host plant neighborhoods.

**Abstract:**

Nutritional content of host plants is expected to drive caterpillar species assemblages and their trait composition. These relationships are altered by tree richness-induced neighborhood variation and a seasonal decline in leaf quality. We tested how key functional traits related to the growth and defenses of the average caterpillar hosted by a tree species are shaped by nutritional host quality. We measured morphological traits and estimated plant community-level diet breadth based on occurrences from 1020 caterpillars representing 146 species in a subtropical tree diversity experiment from spring to autumn in one year. We focused on interspecific caterpillar trait variation by analyzing presence-only patterns of caterpillar species for each tree species. Our results show that tree richness positively affected caterpillar species-sharing among tree species, which resulted in lowered trait variation and led to higher caterpillar richness for each tree species. However, community-level diet breadth depended more on the nutritional content of host trees. Higher nutritional quality also supported species-poorer but more abundant communities of smaller and less well-defended caterpillars. This study demonstrates that the leaf nutritional quality of trees shapes caterpillar trait composition across diverse species assemblages at fine spatial scales in a way that can be predicted by ecological theory.

## 1. Introduction

Herbivorous insect abundance and species richness tend to increase with increasing plant richness [1,2]. Besides plant richness per se, changes in the plant composition may influence the herbivore fauna through plant functional traits [2,3], of which leaf nutritional quality is of especially high importance [4,5].

The Resource Concentration Hypothesis [6] predicts that specialists concentrate in patches of low plant richness based on resource abundance. In turn, higher plant richness may offer possibilities for dietary mixing for herbivores [7,8], and some studies have demonstrated such nutrient-balancing behavior to be stronger with generalist than specialist herbivores [9,10] (but see [11]). Other ecological factors than balancing nutritional intake are, however, likely also to lead to mixed diets [8]. For example, the Optimal Foraging Hypothesis [12] posits that herbivores make diet choices to optimize energy, nutrient, and time demands [13,14]. Higher plant richness can then enable host plant shifts through increased options in the host plant neighborhood. 

Caterpillars represent a substantial component of total insect diversity in forest ecosystems [15] and are a common model group in nutritional ecological studies (e.g., [10,16,17]). Host plants are selected both by ovipositing female Lepidoptera and by caterpillars [18]. Caterpillars can switch among host plants either through ballooning (via silken thread) or by locomotion [19,20]. Through host plant shifts, caterpillars can offset poor oviposition choices [18,21], balance nutritional intake [22,23,24], shift from the high protein demand of early instars to the high carbohydrate demand of later stages [9,25], and increase food resources (e.g., feeding habit shift from miner to external feeder [26]). Conversely, caterpillars may be directed towards suboptimal food sources in order to escape parasitism and predation through host plant shifts [27,28,29]. 

The positive effect of high nutritional quality on herbivorous insect preference has been demonstrated to be strong [5]. Nitrogen (N) especially is a major limiting factor in the growth of herbivorous insects [4,30], and the lower use efficiency of generalists can lead them to favor high N diets or to rely on over-ingestion [24,31,32]. Required carbohydrates can also be limiting, as carbon (C) is largely present in less usable structural carbohydrates and as digestibility-reducing and feeding-inhibiting tannins [30,31]. Tougher, high C-content plant material can affect the caterpillar feeding traits by requiring stronger head musculature [33,34,35] or by selecting for smaller species or individuals that can selectively consume the more palatable portions within the leaf (reviewed in [36]). C content increases while N often decreases with leaf maturation [4,17,37] (but see [38]). A similar pattern follows for leaves produced later in the season [39], and these changes can have profound effects on caterpillar growth and defenses [16]. While more nutrient-rich, early-season leaves increase the growth rates of caterpillars [16,39], they have been found to particularly favor smaller herbivore species [4,40]. A decrease in content by leaf age has also been shown for other nutrients, such as magnesium (Mg), phosphorus (P), and potassium (K) [38,41,42] (but see opposing results for Ca [43]). N, P, K, Mg, and Ca are all essential nutrients for herbivorous insects [35,44], and micronutrients, such as Mg, have been shown to modify caterpillar species composition [45] and amplify the effect of macronutrients (N, P, K) on arthropod abundance [46]. Mg, specifically, is an important element in hemolymph, cuticle formation, and tissues, including the nervous system [31,44,47]. However, leaf nutrients other than N have received much less attention in nutritional ecology studies so far. 

Leaf traits and plant species richness can also influence caterpillar traits that are indirectly linked to resource use. Plants or older leaves with lower nutritional quality can reduce the growth rate of caterpillars and, thus, prolong development time and increase their vulnerability to predation, which, in turn, may demand greater investment in defense mechanisms [4,16,48]. Hairs offer physical repellence against predators [49,50], and coloration can work as a defensive trait through camouflage and aposematism (e.g., [49,51]). Higher conspicuousness in later instars can promote aposematism due to increased size and mobility [51,52]. Aposematism can also be more useful in later seasons outside the naïve fledgling period [53]. Increasing plant richness may promote predator abundance (the Enemies Hypothesis [6]), which may lead to a higher need for defensive traits. However, empirical knowledge on how plant richness, leaf traits, and seasonal change together influence the caterpillar community via the caterpillars’ body size, diet breadth, and defensive traits is still lacking.

In this study, we aimed to test how the leaf nutritional content of trees affects the trait composition of caterpillar communities and how this relationship is altered by the surrounding tree richness and seasonal progression in a subtropical tree diversity experiment. We tested what kind of caterpillar fauna, on average, a tree species hosts at a given point in time, along with increasing tree neighborhood richness, by sampling over spring, summer, and autumn during a single year. Presence-only sampling units for trait analysis were formed by averaging the caterpillar traits across species for each tree species per tree richness level per season. Changes in trait variation were tested as functional diversity of caterpillar fauna within each tree species and between the caterpillar trait averages among tree species. We expected that: (i) tree richness increases sharing of caterpillar species between tree species (measured as tree richness level–specific beta diversity), which also shows as increased caterpillar generalism (measured as occurrences between all sampling units) and as an increase in caterpillar richness per each tree species; (ii) increased caterpillar species-sharing among tree species at higher tree richness results in reduced caterpillar trait variation. Similarly, it reduces within-tree species functional diversity (functional dispersion, FDis); (iii) higher nutritional content (N and Mg), which is promoted in early seasons, leads to lower body weights by favoring earlier instars and smaller species, and lower defensive traits due to faster development. Carbon content (C) is expected to have the opposite effect of N and Mg on body weight due to tougher and palatability-reducing structural carbohydrates and tannins, but, alternatively, can lead to increased growth rates because of the higher amount of shorter, more usable carbohydrates. Similarly, the effect of C on head size may be positive or negative, either by promoting reduced head size and selective feeding or by increasing head musculature. By linking tree richness and leaf nutritional traits, and the functional composition of a highly diverse herbivore larval community in a controlled tree richness setup, our study provides insights into how bottom-up effects shape caterpillar communities through fine-scale habitat selection.

## 2. Materials and Methods

### 2.1. Study Region and Experimental Design

This study was carried out in the subtropical region of southeast China (Jiangxi Province, 29°08′–29°11′ N, 117°90′–117°93′ E, 105 to 275 m above sea level) as part of the BEF-China biodiversity experiment. BEF-China is currently the largest tree diversity experiment in the world, where tree richness and tree species composition of individual plots were manipulated following a strict design (see also map of the area [54]). The study area used was site A of the BEF-China experiment, which has a stand-alone tree diversity setup planted in 2009. The 26.7 ha study site comprises 271 plots, of which 69 were used in this study. The plot size is 25.82 m × 25.82 m, which corresponds to the traditional Chinese unit of 1 mu (666.7 m^2^). Within each plot, 400 trees were planted in a 20 × 20 (rows by columns) design. The selection of species followed a random broken stick design for extinction scenarios with mixtures of 24, 16, 8, 4, and 2 species and monocultures. The 24-species mixtures are an additional treatment on top of the gradual extinction scenario design starting from the 16-species mixture [54]. The richness levels 16 and 24 were combined to form a single ‘high richness’ level that was named ‘16’ in the analyses and results and used in order to avoid crowding tree replicates closely together (fewer plots towards higher richness levels as tree species appear on same plots). From the species pool involving 24 tree species, 16 were used in this study. The species used in the study consisted of deciduous species: *Castanea henryi* Rehd. and Wils., *Choerospondias axillaris* Roxb., *Koelreuteria bipinnata* Franch., *Liquidambar formosana* Hance, *Nyssa sinensis* Oliver, *Quercus fabri* Hance, *Quercus serrata* Murray, *Rhus chinensis* Mill., *Sapindus mukorossi* Gaertn., and *Triadica sebifera* L.; and of evergreen species: *Castanopsis eyrie* Champ. ex Benth., *Castanopsis sclerophylla* Lindl. and Paxt., *Cyclobalanopsis glauca* Thunb., *Cyclobalanopsis myrsinifolia* Blume, *Lithocarpus glaber* Thunb., and *Schima superba* Gardner and Champ. No living individuals of *Rhus chinensis* Mill. were found in 2-species mixtures, so these plots were reassigned as *Schima superba* Gardner and Champ. monocultures. Some individuals of *Castanopsis eyrei* Champ. were recognized to belong to *Castanopsis fargesii* Franch. and were excluded from the data. The number of sampled trees per species per richness level varied from 1 to 16 with an average of 5.9 (SD = 2.5) (see Table S1 for all tree replicate numbers). The replicate numbers vary to some extent, besides tree species and richness level corrections, due to self-thinning making some trees very rare in certain richness levels. Moreover, we followed a sampling scheme that matches the sampling of leaf traits [55], which increases the intended tree replicate numbers per tree richness level from 5 to 6 in monocultures and to 9 in 2-species mixtures. Effects of differences in tree replicate numbers were accounted for in the analyses by using replicate numbers as a covariate in caterpillar richness analyses (see Section 2.5.1). 

### 2.2. Sampling Strategy

The caterpillar samples were collected three times in 2019 (April–May, June–July, and August–September), from 449 tree individuals in spring, 465 in summer, and 463 in autumn, with the campaign lasting 12, 16, and 16 days, respectively. Caterpillars were collected by beating the tree crown seven times with a padded stick over a suspended white sheet (1.0 m × 1.0 m) that was lifted as close as possible to the branches under collection by a telescopic pole reaching a maximum of 8 m height. Due to this collection method, the caterpillars represent only external feeders. All dislodged caterpillars were collected and stored separately in tubes with 99.5% ethanol.

Given the throughput limitations in the morphological identification of caterpillars, DNA barcodes were sequenced from all caterpillar individuals and clustered into Molecular Operational Taxonomic Units (MOTU, hereafter referred to as species) for further analyses. Threshold-based hierarchical clustering with BLASTclust [56] (threshold of 97.8% identity [57]) was adopted as the species delimitation method following the pipeline of [58] due to the threshold-based method’s conservatism with datasets consisting of many singleton sample species, which can lead to poor performance of variance-based methods [59]. 

One sample was excluded from the data, as leaf traits were unavailable for *Koelreuteria bipinnata* Franch. for the high richness level. One sample was recognized to have an obvious measurement or recording error in weight and was excluded. Finally, 57 caterpillars were removed because of inability to measure some trait values due to tissue damage. After these exclusions, 1020 caterpillar–plant interactions (i.e., caterpillar) samples were retained for subsequent analyses.

### 2.3. Caterpillar Trait Measurements

We measured the diet breadth of each caterpillar species as occurrences across all tree species within the plant community (generalism). Body weight, head capsule width, hair coverage, and aposematism were measured from each caterpillar individual. Body weight (with an accuracy of ±0.1 mg) was measured from the ethanol-stored caterpillars first air-dried on Petri dishes. Head capsule width was measured under a stereoscopic microscope with a measuring scale with 10× to 40× magnification. An increase in head capsule width is highly correlated with an increase in body weight. In addition, head size may be hypoallometric (decrease relatively as the body grows) because body weight increases continuously during the instar development, but the sclerotized head capsule size increases when molting and is assumed to follow a geometric growth ratio along instars (named Dyar’s rule [60,61]). The difference in head and body size is expected to be most pronounced at the end of each instar prior to molting [62]. For this reason, in addition to using body weight as a covariate in the head capsule width analysis (see Section 2.5), relative head capsule width was calculated (head capsule width divided by body weight). Further, the body weight (mg) values were cube root-transformed to increase linearity and correlation to the one-dimensional head capsule width (correlations of original and cube root transformed values shown in Figure S1). 

**Figure 1:**
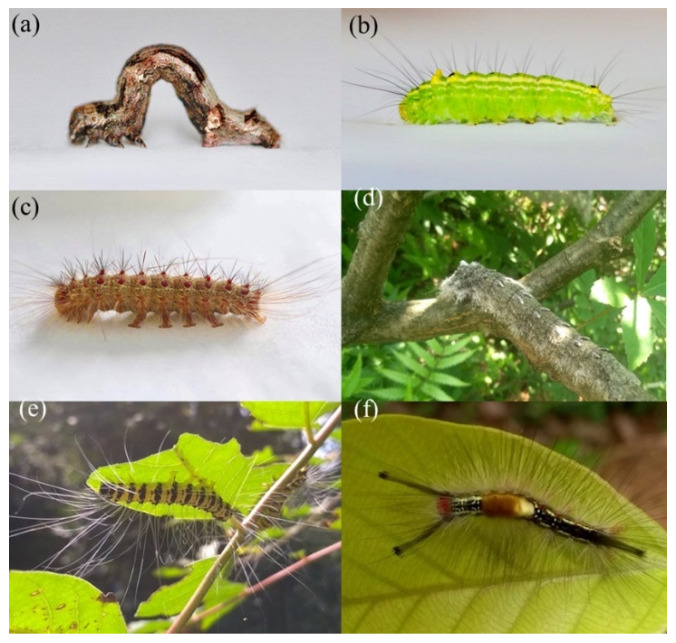
Hair coverage and coloration categories employed in this study, presented by example species encountered in BEF-China: (**a**) 0, (**b**) <25%, (**c**,**e**) <50%, and (**d**,**f**) >50% hair coverage. Pictures (**a**–**d**) represent camouflage, and (**e**,**f**) aposematism. Photo credit: (**a**–**c**) Y. Li, (**d**–**f**) P. Anttonen.

Hair coverage and aposematism were evaluated by using similar methods to those in other studies of caterpillar defenses, e.g., [16,27], but with adding one more class to hair coverage evaluation (<25%) and simplifying the aposematism to two classes instead of three (Figure 1), as compared to methods in [16]. The hair coverage was estimated visually under a stereomicroscope and it informs how much of the caterpillar cuticle is covered due to the combination of hair density and hair length. Hair coverage was classified into four levels: 0 (Figure 1a), 1 (<25%; Figure 1b), 2 (<50%; Figure 1c,e), and 3 (>50%; Figure 1d,f). The color of the caterpillars was visually recorded as the coloration that covers at least 80% of the body surface (mainly excluding the head). Observed colors varied from black to light yellow and were divided into camouflage (singular colors, e.g., green, grey, and black) (Figure 1a–d) and warning colors (bright coloration and high contrasts such as bright yellow and red, and black and yellow stripes) (Figure 1e,f). The color classification was intentionally robust, focusing on the color contrasts rather than specific color shades to account for potential fading because of storing the samples in ethanol.

### 2.4. Leaf Trait Selection

Specific leaf area (SLA), leaf dry matter content (LDMC), nitrogen (N), carbon (C), magnesium (Mg), calcium (Ca), potassium (K), and phosphorus (P) were measured on all trees used in the study (details on leaf trait measurements are provided in Supplement S1; see also [55,63]). The selection of leaf traits from candidates for analysis was based on internal collinearities (Pearson r < 7; see Figure S1 for trait correlations). Many nutritional leaf traits were highly correlated with each other: N and Mg with K and P, and Mg also with Ca. For this reason, N and Mg were considered the most suitable overall estimators of nutritional quality. C content was also included for further information on leaf quality. The structural leaf traits SLA and LDMC were not included in analyses because of their high correlation with each other, and of SLA with N and LDMC with C. Leaf samples were collected from multiple tree individuals per tree species between August and October 2018 and comprised only fully developed, non-senescent leaves free of damage from herbivores, pathogens, or mechanical stress. Leaf traits were averaged for statistical analyses for each tree species per tree richness level in order to account for neighborhood richness-induced changes in leaf traits [55,64].

### 2.5. Statistical Analyses

#### 2.5.1. Caterpillar Trait Data Processing and Linear Analysis

**Figure A1:**
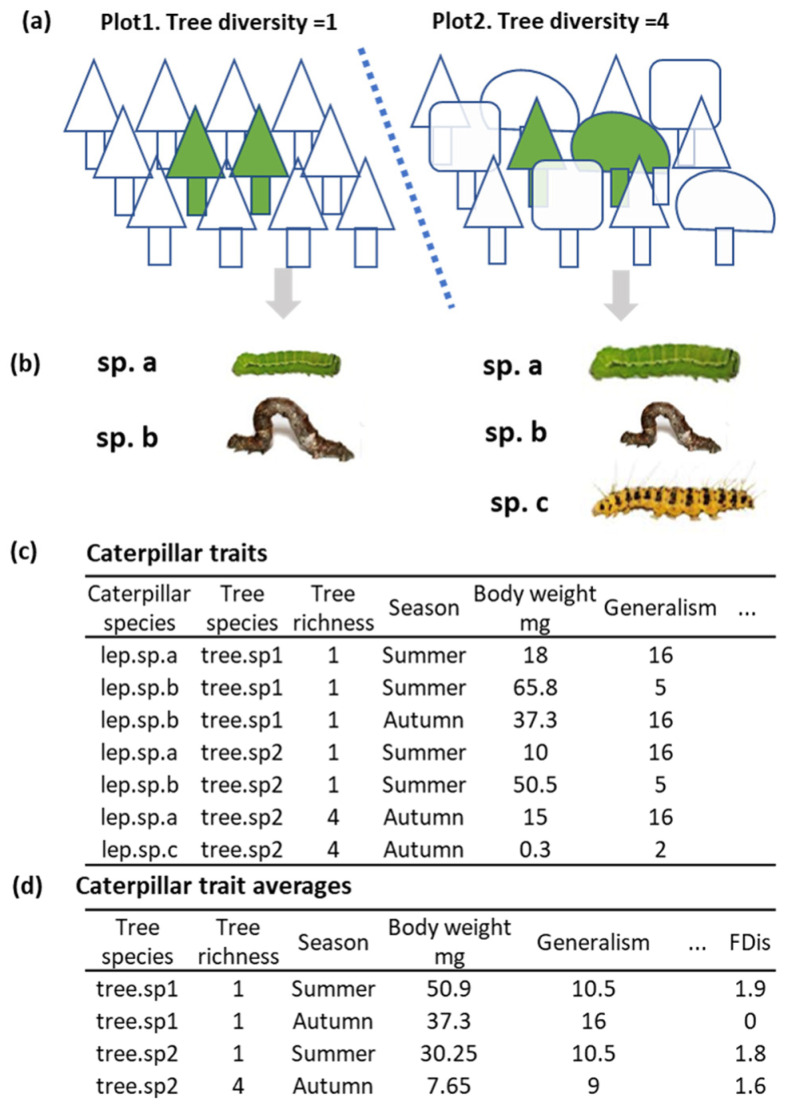
Depiction of the general framework of this study: (**a**) monoculture shown on the left and 4-species mixture on the right; (**b**) caterpillars collected from these tree richness levels during two seasons; (**c**) caterpillar trait averages per caterpillar species per tree species per tree richness per season; (**d**) caterpillar trait averages across caterpillar species per tree species per tree richness per season, as used in subsequent analyses. Averaging of caterpillar values per caterpillar species first per tree individuals not demonstrated for brevity. FDis values were estimated between all traits using caterpillar species averages for each tree species per tree richness level per season.

In order to focus on interspecific variation, the study sampling unit was formed by averaging caterpillar intraspecific trait values, followed by interspecific averaging for each tree species and season (see Figure A1 for the process pipeline of the study). Intraspecific caterpillar trait values were first averaged for each caterpillar species per tree individual to account for differences in traits caused by solitary and gregarious individuals of different group sizes [65], followed by averaging between tree individuals per tree richness level per season.

The averaged caterpillar data were analyzed using linear mixed-effects models using package lmerTest [66] in R v 4.0.5 environment [67]. Response variables used were: caterpillar species richness, abundance, generalism, body weight, head capsule width, relative head capsule width, aposematism, and hair density. All caterpillar trait models were analyzed using tree richness, sampling season, leaf traits (N, Mg, and C), and two-way interactions of tree species richness and season with each other and leaf traits as explanatory variables. Tree species identity was used as a random factor in all trait models. The number of replicates for each tree species per richness level was included as a covariate for caterpillar species richness and abundance models. Species richness was used as a covariate for caterpillar trait analyses to account for the directional effect caused by lower caterpillar species numbers on specific tree species. Because the likelihood of being caught increases by the number of tree species a caterpillar species occurs on, no exclusions of rare species (based on abundances) in the data were performed in order to not disproportionately weight the effect of generalist species. The correlation of body size to other caterpillar traits was tested by including body weight as a covariate. The correlation of defensive traits (aposematism and hair density) was tested by including them in each other’s models.

All models were reduced using backward selection with function ‘step’ in the package lmerTest [66] to obtain the most parsimonious model. All fixed factors, but not the random factors, were allowed to be dropped from the model with selection criterion *p* < 0.05. All predictors were tested for collinearity with Pearson’s correlation r > 0.7 (Figure S1). Variance inflation factors (VIFs) were calculated using the package car [68] to ensure no strong collinearity among the predictor variables (all VIFs < 5; see also [69]). In order to improve the normality and variance homogeneity of the model residuals, leaf trait values and all response variables were log (x + 1)-transformed. The cube root transformed body weight values were used in all analyses to increase linearity.

Single factors in linear mixed effect models can have significant F-test values but improve the overall information value of the model only marginally, or even increase the AIC value by increasing the number of explanatory variables. In order to evaluate whether leaf traits and other factors of interest improve the overall models, random variable- and covariate-only models were run for each model with (1) only tree species identity as a random variable and (2) with the following non-hypothesis covariates: tree replicates for caterpillar species richness, tree replicates and caterpillar species richness for abundance, caterpillar species richness and body weight for head capsule width, and only caterpillar species richness for the other trait models and FDis (see below). All models were evaluated by comparing the AIC-values (Akaike Information Criterion value) [70] against the random variable- and covariate-only models, with ΔAIC > 2 interpreted to offer an improved model.

Further, sensitivity comparisons of the effect of the most common and widely spread caterpillar species between tree species were conducted. This was conducted for all caterpillar traits and FDis by removing all species that appeared on more than half of the tree species. This accounted for 11 species with a total of 621 individuals (~60.6% of all samples).

#### 2.5.2. Caterpillar Functional Diversity within Tree Species

Functional dispersion (FDis), which works without abundance-weighing as a measure of functional diversity [71], was used for measuring within-study unit caterpillar functional diversity. Caterpillar FDis was calculated based on caterpillars’ body weight, head capsule width, hair coverage, aposematism, and generalism. Increasing FDis values indicate higher overall trait variation around the trait centroid [71]. The abundance of each species was set as one to focus on the effects of interspecific trait variation. The change in FDis between sampling units was analyzed using tree richness, sampling season, leaf traits, and interaction between them as explanatory variables, and caterpillar species richness was included as a covariate.

#### 2.5.3. Caterpillar Trait Variation and Species-Sharing between Tree Species

In addition to functional trait diversity within tree species, the caterpillar trait variation between tree species was estimated by calculating the standard deviation (SD) of the caterpillar trait values between sampling units. The change in standard deviation was analyzed with linear models with the function ‘lm’ in the R environment. Explanatory variables used were tree richness, sampling season, and caterpillar species richness. Body weight averages for the respective tree richness levels were also included in the relative head capsule width model to test if systematic changes in body weight lead to increasing variation in the head capsule width. 

Caterpillar beta diversity between tree species for each tree richness level was estimated for comparison to caterpillar trait variation between tree species and species richness per tree species. The change in community assembly was tested using Sørensen dissimilarity and, separately, its two components, species turnover using Simpson’s dissimilarity and nestedness, with the R package ‘betapart’ [72]. These richness level-specific values were tabulated and analyzed with the function ‘lm’ in the R environment. Explanatory variables used were tree richness, sampling season, and the number of sampled plots per richness level in order to account for decreasing spatial variation towards higher tree richness (more monoculture plots than species mixture plots). Due to the low number of data points in the between-tree species analyses (15), no interactions between explanatory factors were included.

#### 2.5.4. Caterpillar Intra- and Inter-Specific Trait Variation

Because caterpillar traits can vary both between and within species, a robust comparison of intra- and inter-specific trait variation was conducted in order to test their relative effect on the underlying trait patterns. Overall, caterpillar intra-specific trait variation was estimated for each caterpillar trait as the average of standard deviations (SD) of each species that had more than one individual. The inter-specific variation was estimated as the standard deviation between caterpillar trait averages of each caterpillar species across all sampling units.

## 3. Results

In total, we analyzed 1020 successfully sequenced and trait-evaluated caterpillar samples. Sequencing failed for 15 samples of 1092 caught. The caterpillars were delineated to 176 species by BLASTClust, of which 146 were retained for further analysis after the exclusion of missing trait values and incorrect tree species. Caterpillar species richness and abundance with respective tree replicates for each sampling unit are shown in Table S1. In total, caterpillar species were assigned to 17 Lepidoptera families, in which Erebidae (48 species, 309 individuals) was the most species-rich and Geometridae (33 species, 573 individuals) the most abundant (Table S2). Other families had much lower species numbers and abundances, with the third most species-rich family (Notodontidae) having 11 species and an abundance of 22, and the third most abundant family (Sphingidae) having 2 species and an abundance of 33. Around 17% of the caterpillar species (25 species, 33 individuals) could only be assigned reliably to the rank of order (Lepidoptera) and thus were labeled as unassigned Lepidoptera.

### 3.1. Caterpillar Traits

#### 3.1.1. Diversity and Generalism

**Figure 2:**
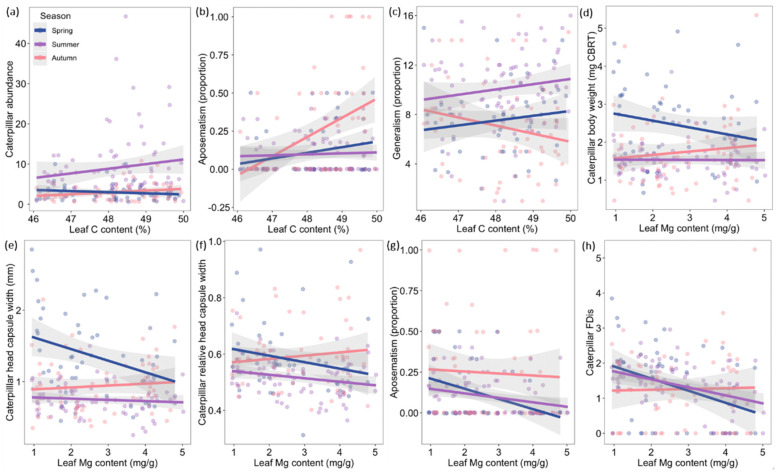
Relationships between leaf traits (leaf C and Mg content) with caterpillar (**a**) abundance and traits, (**b**) body weight, (**c**) head capsule width, (**d**) relative head capsule width, (**e**,**f**) aposematism, (**g**) generalism, and (**h**) FDis.

Caterpillar species richness was positively affected by the number of tree replicates and tree richness and negatively by leaf Mg content (see Table 1 for full model results). Abundance was highest in summer (693), as compared to spring (150) and autumn (177), and was best explained by increasing caterpillar species richness. Abundance also increased with increasing leaf Mg and with the interaction of leaf C content and season (Figure 2a), with increased abundance in high C content in the summer.

**Figure 3:**
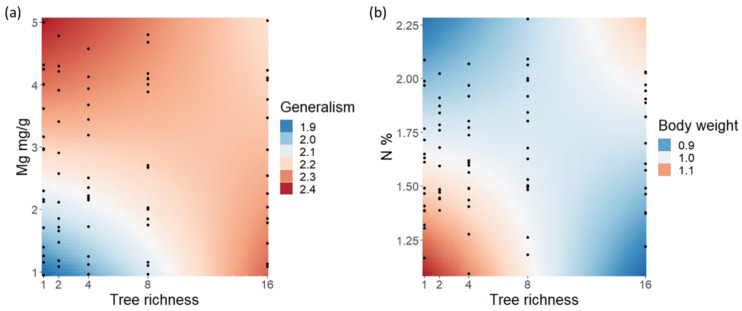
Relationship of caterpillar traits to interaction of leaf traits and tree richness. Color gradient represents estimated change in response values in the lme models retrieved by package ‘effects’ [68], and point clouds show observed values. (**a**) Change in generalism through interaction of tree richness and leaf Mg content, and (**b**) caterpillar body weight change through interaction of tree richness with leaf N content. All factors except tree richness are log + 1 transformed. Body weight values are also cube root transformed.

Generalism of caterpillar species varied between 1 and 16 host tree species, but with the vast majority of species being found only from one (92 species) or two (20 species) tree species (Table S3). Average generalism between sampling units was 8.42 (SD = 3.56). Generalism per tree species was positively correlated with caterpillar species richness and promoted by high leaf Mg content (see Table 1 for full model results). Tree richness showed only a weak direct positive effect on generalism, but the negative effect in low richness was stronger with low Mg content, with little to no effect of Mg in high richness (Figure 3a). A negative effect for generalism was shown by body weight and season, with the latter especially, in high C-content tree species (Figure 2c). However, C content showed a positive trend for generalism in spring and summer.

#### 3.1.2. Growth-Related Traits

Body weight was higher in spring and especially in low Mg content tree species that, in turn, hosted slightly smaller caterpillars in autumn (Figure 2d) (see Table 1 for full model results of all growth-related traits). Nitrogen and tree species richness had only a weak negative overall effect on body weight, but the negative effect of N in the low tree richness levels was stronger (Figure 3b). 

Head capsule width increased strongly with increasing body weight and decreased with increasing C, N, and Mg content. The negative effect of Mg was especially strong in spring, with a weak positive trend in autumn (Figure 2e). Head capsule width was also negatively correlated to caterpillar species richness.

The relative head capsule width was negatively affected by body weight and caterpillar species richness. Relative head capsule width decreased with increasing Mg and its interaction with the season (Figure 2f), with a negative effect of increasing Mg in spring and summer and a positive effect in autumn.

#### 3.1.3. Defensive Traits

Hair coverage and aposematism were strongly positively correlated with each other (see Table 1 for full model results). C content had a negative effect on hair coverage, and a positive effect on aposematism, with the strongest effect in autumn (Figure 2b). Increasing N content also had a negative effect on hair coverage. Hair coverage was reduced in later seasons, and aposematism increased. Mg content had an overall negative effect on aposematism in spring and summer, with the overall higher autumn values being affected only slightly (Figure 2g). Body weight had a positive effect on aposematism.

#### 3.1.4. Functional Diversity within Tree Species and Sensitivity Comparison

FDis of the caterpillar traits was strongly positively affected by caterpillar species richness (see Table 1 for full model results). Mg content had a negative effect on FDis in spring and summer (Figure 2h).

Our sensitivity comparison, with the most common species removed, led to similar overall results but with several of the weaker explanatory factors being dropped (Table S4). Additionally, in the case of relative head capsule width, seasonal interaction with Mg was replaced with tree richness interaction with Mg, and C content showed a negative effect. All random variable- and covariate-only models had a clearly worse fit (higher AIC values) than the fixed effect predictor models. Interspecific variation (SD) of caterpillar trait values was about twice as large as intraspecific variation, except for relative head capsule width, where they were nearly equal, and aposematism and hair coverage, which had no intraspecific variation (Table S5).

### 3.2. Functional Trait Variation and Species Sharing between Tree Species

Increasing caterpillar species richness had a positive effect on variation of abundance, and a negative effect on other traits, with significant trends on body weight, head capsule width, hair coverage, aposematism, and FDis (see Table A1 for all SD model results). Tree richness had a strong negative effect on SD of head capsule width, a marginally significant negative effect on hair coverage, aposematism, and FDis, and a moderately negative trend on body weight. Season had a positive effect on SD of generalism and aposematism. None of the explanatory factors had a strong effect on variation of caterpillar species richness and relative head capsule width.

Beta diversity was more strongly driven by species turnover than by nestedness (see Table A2 for linear model results of changes in beta diversity). The number of sampled plots showed only a weak negative trend on caterpillar species turnover and nestedness but moderately stronger on beta diversity. Tree species richness had a strong negative effect on beta diversity and species turnover and a moderately positive trend on nestedness. Season also had a strong positive effect on beta diversity and species turnover and a moderately negative trend on nestedness.

## 4. Discussion

By using functional traits of leaves with corresponding traits of herbivores (caterpillars) in a tree diversity experiment with controlled tree richness and freely assembled insect fauna, our study provides insight into how functional traits determine interactions between consumer insects and primary producers through bottom-up effects. Our study answers what kind of caterpillar fauna, on average, is observed in tree species with varying nutritional quality at a given point in time along a tree richness gradient, and how tree richness affects caterpillar trait variation between the tree species. Caterpillar traits, especially body size, may change along the caterpillars’ development, and it should be noted that the scope of the study is limited to comparisons between tree species at specific time points without extending to what ultimate body sizes or other trait values would be reached by specific caterpillar species on variations of diets. However, seasonal and body size-dependent trait patterns show that caterpillar functional traits are dynamic and deserve consideration beyond simple species-to-species connections. The tree richness gradient and leaf traits in this study reflect habitat quality at fine spatial scales, enabling a better understanding of how local conditions shape the community assembly of the caterpillar fauna via trait matching.

### 4.1. Tree Richness Effects on Caterpillar Trait Composition

The direct effects of tree richness on caterpillar functional traits remained weak in our study. This is in concordance with earlier results from the same field site, which demonstrated the higher importance of tree functional and phylogenetic composition on herbivore assemblage [73]. Nevertheless, there was a systematic negative trend on between tree species variation across caterpillar traits and also a weakened effect of N on body weight towards higher richness levels. However, no strong trends of tree richness were observed for within-tree species caterpillar trait diversity (FDis). In a separate study from the same field site [74], higher caterpillar species co-occurrences between tree species with higher nutritional quality were found. Here, we found particularly strong negative effects of tree richness on caterpillar beta diversity among tree species, which likely explains the accompanied reduced trait variation and the interactive effect of tree richness with leaf traits. The increased caterpillar species-sharing between tree species can also explain the increased caterpillar richness for each tree species (i.e., at the tree species level) in higher tree richness, in combination with plot-level increases, shown in a previous study for the field site [58]. These results differ from what has been observed at wider spatial scales, where it has been shown that the increase in herbivore species richness across latitudinal gradients can be a direct cumulative effect of increasing plant species richness with no plant species-level increase [15]. Studies on trait variation between separate field sites, and thus presumably different herbivore communities, have also shown increases along increasing plant richness [75,76,77]. Thus, our results suggest that the increasing variation of host plants is not necessarily accompanied by increasing trait variation of herbivores at fine spatial scales within the herbivore community if it leads to more mixing of the fauna and highlights the need to take into account the spatial scale in trait studies.

For our fine spatial-scale approach, the decrease in beta diversity at higher tree richness can be seen as support for the Resource Concentration Hypothesis [6], stating that higher plant richness favors generalists. However, direct effects on our site-specific generalism measure were weak, despite an earlier study in the same field site showing positive effects of tree richness on generalist abundances at the sapling stage [78]. Effects of tree richness on generalism became more evident with leaf trait interactions, specifically in relation to leaf Mg content, where the overall effect of Mg on generalism was positive and further promoted in lower tree richness levels. This nutrition–tree richness connection demonstrates that the level of herbivore generalism a tree species experiences is a combination of the trait variation in the tree neighborhood and a tree species’ nutritional quality, with the latter appearing to be relatively more important. The caterpillar species-sharing between tree species can be a result of more widely spread oviposition, increased movement of caterpillars within vegetation, or a mixture of both. As caterpillar chemoreception capabilities are limited [79] and movement in vegetation is essentially non-targeted, generalists, with their lower nutrient use efficiency [24], are more likely to benefit from dietary mixing opportunities in the immediate surrounding vegetation. It should be noted, however, that the high number of species found only on single tree species in our study are unlikely to represent only strictly monophagous species, which can be rare even in diverse tropical insect communities [80,81]. Nonetheless, as the local plant community determines the possible diet breadth for herbivores, looking at the diet breadth within the plant community makes a realistic estimate of herbivore–plant interactions at fine spatial scales [82]. Such a community perspective allows looking into habitat selection through nutritional differences represented by the dominant plant species of a local community.

Whether higher plant richness increases predation pressure (Enemies Hypothesis [6]) has been debated due to confounding plant structural, predator, and parasite intra-guild interactions [83,84]. We did not observe directional effects of tree richness on defensive traits but found a negative trend in trait variation. As nutritional content was observed to have a strong effect on defensive traits, the lack of directional effects of tree richness does not necessarily mean a lack of top-down pressure but a possible interference effect on optimal defense strategies in highly variable environments. Notably, generalist species have been shown to be less well-defended than specialists [85].

Overall, how herbivorous insects are affected by tree richness has focused more on the important role of plant defenses [86] than on nutritional content. Our study suggests that tree richness not only has bottom-up effects on herbivorous insects through nutrition, but may also subsequently alter the top-down effects they experience. In this sense, tree richness, plant defenses, and nutritional quality should be viewed together in future studies of herbivore community assembly.

### 4.2. Nutritional and Seasonal Effects on Caterpillar Traits

Unexpectedly, our results showed overall higher effects of leaf Mg on caterpillar abundance and trait composition than leaf N content. Some studies have demonstrated the importance of micronutrients such as Mg [45,46] on herbivore insects, but most of the research with caterpillar dietary requirements has focused on the balance between protein and carbohydrate intake (e.g., [23,24,25]). However, if generalists possess overall lower nutrient use efficiencies, as shown in an example case for N [24], and also indicated by slower growth rates compared to specialists in a multi-species experiment [16], it would be a valid strategy to also favor other essential high nutrient concentrations and balance the overall intake [14]. This interpretation would correspond to the expectations of the Optimal Foraging Hypothesis [12,13].

Nutritional effects did not remain stable over the seasons. Even though a reduction in leaf quality towards later seasons is expected to be common among plant species [4,38,41], using season alone as a proxy for leaf quality poses some problems due to differences in the base level of the leaf nutritional content between tree species. Other factors, such as predation pressure, can also vary between seasons [53,87], obscuring the mechanisms through which the seasonal progression affects the caterpillar community. Increased body size of caterpillars in later seasons has been attributed in some studies to reduced predation and overwintering preparation [62,87], but with simultaneously increased leaf age leading to higher dispersal, presumably due to escape from poor quality hosts, and earlier populations also having higher fecundity [62]. In addition, smaller herbivorous insects may prefer younger leaves [4,40], and caterpillar generalism has been shown to be higher in spring [58,88]. The higher body weights we observed in spring question the uniformity of these responses. However, assuming smaller insects prefer younger leaves because of higher nutritional content, our results—with lower body weights in spring with increasing Mg content and higher N content in monocultures—fit well in the framework. The tree richness-dependent effect of N content may demonstrate the easier finding of host plants by smaller, high nitrogen content-favoring species. Alternatively, or in addition, the negative N–body size relationship may be explained by a higher proportion of earlier instars that benefit from higher N content [9,25].

In our study, hair coverage was connected to lower nutritional content, similar to aposematism, promoting the interpretation that lower nutrient content leads to slower growth rates and a higher need for defenses [4,48]. Interestingly, hair coverage was higher in spring as opposed to aposematism, even though the two traits were correlated, suggesting otherwise a defensive trait syndrome. A possible explanation for the disparity of hair coverage and aposematism in regard to season comes from the effect of predation by birds. Aposematic coloration is expected to be of higher importance outside the fledgling season [53], and hairiness could be an alternative defensive measure in spring by not suffering from increased conspicuousness.

Additionally, beta diversity and variation of generalism and aposematism between tree species increased towards autumn. This, along with generalism being more common in spring and aposematism in autumn, shows the species pool was more strongly separated along these traits when the differences between tree species in leaf nutritional content are expected to be highest. Similarly, within-tree species FDis was also negatively affected by nutritional content (Mg) in spring and summer, demonstrating the unifying effect of nutritional content on the caterpillar species pool. In addition, Mg also increased abundance despite reducing species richness. Predation can shape the caterpillar community in low nutrient content, favoring more specialized species if they are better defended [85], but competitive exclusion can also be strong between herbivore species [89,90], and the observed pattern might suggest that generalists have competitive advantages in high nutrient environments.

The observed trends with respect to increasing C content were bi-directional, with, on the one hand, higher aposematism (overall, but especially promoted in autumn) and lower generalism in autumn, which can be expected by higher structural carbohydrate and tannin concentrations on caterpillar growth [17]. However, on the other hand, hair coverage, generalism, and caterpillar abundance were promoted by high C content, with the latter two in earlier seasons, which can be expected by carbohydrates being essential nutrients in the caterpillar diet [22,31]. In addition, older instars can shift their preference from a protein-rich to a carbohydrate-rich diet [9,25]. However, where we observed negative effects of nutritional content on body weight by N and Mg, we did not observe any positive relationship of C. Instead, head capsule width was negatively influenced by C content, which is according to the expectation of smaller individuals eating selectively from tougher leaf tissue [36]. However, the effect of N and Mg was similarly negative on head capsule width, even after accounting for the effect of body weight and thus the promotion of larger amounts of early instars, which obscures what role the C content and nutrition in general play in determining the head size of caterpillars. Moreover, tougher food content has, in other cases, been connected to increased head sizes [33], and it may be that the head size–C content relationship is dependent on the instar stage, as most caterpillars in this study were very small and presumably early instars. Our results also showed that besides leaf nutritional content influencing the head capsule width, as seen also with (Mg) on relative head capsule width, smaller caterpillars had relatively larger heads, suggesting a hypoallometric relationship of head and body size. The expected geometric growth rate of head size along Dyar’s rule has been shown to weaken during the development of the caterpillar and respond to growth-related factors [61,91,92]. Increasing body weight could then be expected to increase the between-tree species variation of relative head capsule width because of nutritional effects, which, however, was not observed in this study. More light on the nutritional content–head size relationship would be gained by the inclusion of mandible structures that affect the feeding mode and diet choice [33,34,35,36], but which was out of scope in this study. Overall, the seasonal interaction of carbon content is important to take into account with studies focusing on the bottom-up effects of leaf quality on herbivore traits, but further benefits would be gained by also separating the different sources of carbon within the leaves.

### 4.3. Inter- vs. Intra-Specific Trait Variation in Caterpillars

As we analyzed the caterpillar fauna time point specifically, the results are influenced, besides inter-specific variation, by intra-specific variability due to caterpillar trait ontogenetic change. The inter-specific variation was shown to be clearly higher than the intra-specific variation for all traits except relative head capsule width. However, this comparison does not capture all of the ontogenetic effects, as many of the caterpillars were caught only at a certain life stage. Indication of ontogenetic changes was seen with aposematism, as it was more common in larger caterpillars, supporting the expectation of their higher visibility due to body size itself and the accompanied higher mobility [51,52]. Additionally, Mg content promoted smaller relative head sizes in addition to higher abundances, which hints toward higher proportions of early instars on these trees. In conclusion, inter-specific variation, nevertheless, appeared to be a stronger determinant than intra-specific variation of the observed trait distributions across caterpillar taxa already before intra-specific averaging of the traits.

### 4.4. Methodological Considerations

Some method-related correlations unavoidably arose in our experimental design of manipulated tree richness. However, these effects were, in most cases, weaker than those of tree richness, leaf traits, and caterpillar body weight. The models that include leaf traits also had clearly better AIC values compared to random variable- and covariate-only models. In addition, sensitivity comparison revealed that the used presence-only analysis structure balances the strong abundance differences between caterpillar species, though the trends became somewhat weaker with the loss of the most common species. It is not surprising that the number of tree replicates had a strong effect on caterpillar species richness. This demonstrates, in part, the role of asymmetrical self-thinning caused by tree species competition with high planting densities (representing the natural development of the stands [54]). Self-thinning of long-lived plants in arthropod studies can then influence arthropod species richness, which reflects on their trait distributions, as was seen with caterpillar species richness having effects of varying strength on caterpillar traits in this study. The positive effect of caterpillar species richness on variation of abundance is caused by the highly skewed relationship of these variables, as an increase in overall caterpillar abundance resulted largely from more species being found. We accounted for these confounding effects with the stepwise averaged presence-only approach and use of covariates to ensure reliable estimates. Averaging leaf traits per tree species per richness level also accounts for the potential effect tree richness can have on herbivores by inducing changes in leaf traits [64]. As our study approach is time point-specific, especially in spring, the differences in the timing of budburst between tree species may lead to differences in observed caterpillar body sizes. However, no effect of season on body size variation between tree species was observed, and body sizes were altogether higher in spring than in later seasons, indicating that the leaf phenological effect was likely weak. The larval stage used in this study offers benefits over the adult stage for studies of nutritional effects, as nutrient intake is essentially directed to growth without the interference of reproductive input. However, as the developmental stage, growth rate differences, and species size distributions can induce overlapping effects, the use of immature insects in trait research with naturally assembled communities needs further development of the theoretical background.

## 5. Conclusions

Studies investigating bottom-up effects on multiple caterpillar traits at the community level have been sparse (but see [16]) despite the ecological importance of these interactions. Our study demonstrates that the effects of tree richness, season, and leaf traits predictably influence the species diversity and trait composition of immature herbivorous insects in naturally assembled herbivore communities. The influence of plant richness on herbivore traits was shown to influence the species pool already at a fine spatial scale and to differ from the effects observed at wider scales. The study also demonstrates that the effects of nutritional bottom-up effects require still further investigation on plant–herbivore interactions, especially for nutrients other than nitrogen. Testing the functional bottom-up relationships of plant–herbivore interactions across host plant richness levels is highly important, given the strong impact they have on ecosystem functions [93]. This will further advance the general knowledge of biodiversity–ecosystem functioning relationships [94,95].

## Figures and Tables

**Table 1:** Summary results of linear mixed-effects models after fixed factor reduction using backward selection with criterion *p* < 0.05 for the averaged caterpillar traits per tree species per richness level per season. All factors are scaled by subtracting the mean and dividing by standard deviation. All variables except tree replicates, sampling season, and tree richness are log + 1 transformed. Body weight values are also cube root transformed. Standardized parameter estimates (with standard errors, t and *p* values) are shown for the variables retained in the minimal models. *p*-values in bold mean *p* ≤ 0.05, *p*-values of italic mean *p* ≤ 0.1.

	Caterpillar Species Richness			Caterpillar Abundance		
	*Est ± SE*	*t*	*p*	*Est ± SE*	*t*	*p*
(Intercept)	1.382 ± 0.035	39.759	**<0.001**	1.592 ± 0.018	88.861	**<0.001**
Tree replicates	0.178 ± 0.039	4.607	**<0.001**	-	-	-
Caterpillar species richness	-	-	-	0.656 ± 0.018	35.668	**<0.001**
Tree species richness	0.101 ± 0.039	2.610	**0.010**	-	-	-
Sampling season	-	-	-	0.016 ± 0.018	0.863	0.390
Leaf C content	-	-	-	0.030 ± 0.021	1.450	0.149
Leaf Mg content	−0.097 ± 0.035	−2.774	**0.006**	0.044 ± 0.021	2.073	**0.040**
Season: leaf C content	-	-	-	0.056 ± 0.018	3.070	**0.002**
	**Caterpillar FDis**			**Caterpillar generalism**		
	*Est ± SE*	*t*	*p*	*Est ± SE*	*t*	*p*
(Intercept)	0.747 ± 0.025	29.911	**<0.001**	2.164 ± 0.040	53.703	**<0.001**
Caterpillar species richness	0.298 ± 0.026	11.640	**<0.001**	0.098 ± 0.031	3.135	**0.002**
Caterpillar body weight	-	-	**-**	−0.085 ± 0.032	−2.643	**0.009**
Sampling season	−0.004 ± 0.025	−0.149	0.882	−0.092 ± 0.032	−2.890	**0.004**
Tree species richness	-	-	-	0.040 ± 0.030	1.328	0.186
Leaf C content	-	-	-	0.060 ± 0.044	1.359	0.187
Leaf Mg content	−0.048 ± 0.026	−1.885	*0.061*	0.099 ± 0.045	2.216	**0.036**
Season: C	-	-	-	−0.074 ± 0.030	−2.421	**0.017**
Season: Mg	0.063 ± 0.025	2.468	**0.015**	-	-	-
Tree richness: Mg	-	-	-	−0.072 ± 0.032	−2.276	**0.024**
	**Caterpillar body weight**			**Caterpillar head capsule width**		
	*Est ± SE*	*t*	*p*	*Est ± SE*	*t*	*p*
(Intercept)	0.997 ± 0.019	51.275	**<0.001**	0.655 ± 0.007	99.980	**<0.001**
Caterpillar body weight	-	-	-	0.189 ± 0.006	30.412	**<0.001**
Caterpillar species richness	-	-	-	−0.023 ± 0.006	−3.834	**<0.001**
Sampling season	−0.087 ± 0.019	−4.537	**<0.001**	−0.007 ± 0.006	−1.197	0.233
Tree species richness	−0.018 ± 0.020	−0.909	0.365	-	-	-
Leaf C content	-	-	-	−0.019 ± 0.008	−2.394	**0.030**
Leaf N content	−0.033 ± 0.021	−1.551	0.123	−0.020 ± 0.007	−2.641	**0.015**
Leaf Mg content	−0.004 ± 0.021	−0.204	0.839	−0.025 ± 0.008	−3.155	**0.007**
Season: Mg	0.043 ± 0.020	2.178	**0.031**	0.017 ± 0.006	2.950	**0.004**
Tree richness: N	0.040 ± 0.020	1.998	**0.047**	-	-	-
	**Caterpillar relative head capsule width**					
	*Est ± SE*	*t*	*p*			
(Intercept)	0.440 ± 0.005	82.459	**<0.001**			
Caterpillar species richness	−0.010 ± 0.005	−2.082	**0.039**			
Caterpillar body weight	−0.017 ± 0.005	−3.430	**<0.001**			
Sampling season	−0.001 ± 0.005	−0.216	0.829			
Leaf Mg content	−0.012 ± 0.005	−2.298	**0.033**			
Season: Mg	0.014 ± 0.005	2.902	**0.004**			
	**Caterpillar hair coverage**			**Caterpillar aposematism**		
	*Est ± SE*	*t*	*p*	*Est ± SE*	*t*	*p*
(Intercept)	0.565 ± 0.029	19.980	**<0.001**	0.145 ± 0.014	11.245	**<0.001**
Caterpillar body weight	-	-	**-**	0.038 ± 0.015	2.766	**0.006**
Aposematism	0.203 ± 0.030	6.796	**<0.001**	-	-	-
Hair coverage	-	-	-	0.091 ± 0.015	6.430	**<0.001**
Sampling season	−0.142 ± 0.029	−4.883	**<0.001**	0.084 ± 0.015	5.510	**<0.001**
Leaf C content	−0.091 ± 0.033	−2.775	**0.006**	0.054 ± 0.016	3.095	**0.002**
Leaf N content	−0.074 ± 0.032	−2.297	**0.023**	-	-	-
Leaf Mg content	-	-	-	−0.003 ± 0.016	−0.577	0.564
Season: C	-	-	-	0.058 ± 0.017	3.133	**0.002**
Season: Mg	-	-	-	0.043 ± 0.017	2.313	**0.022**

## Data Availability

Caterpillar trait data: available in a publicly accessible repository that does not issue DOIs. These data can be found here: [https://data.botanik.uni-halle.de/bef-china/datasets/657] (accessed on 29 November 2022). Leaf trait data: restrictions apply to the availability of these data. Data were obtained from Andréa Davrinche and Sylvia Haider and are available from the authors at [https://data.botanik.uni-halle.de/bef-china/datasets/648] (accessed on 29 November 2022) with their permission.

## Supplementary Materials

The following supporting information can be downloaded at: https://www.mdpi.com/article/10.3390/insects13121100/s1, Figure S1: Variable correlations; Figure S2: Relative head capsule width sensitivity analysis; Table S1: Sampling details per tree species; Table S2: Lepidoptera samples per family; Table S3: Caterpillar generalism; Table S4: Sensitivity analysis; Table S5: Caterpillar trait intra- versus inter-specific variation.

## Appendix A

**Table A1:** Summary of linear model testing for changes in caterpillar trait standard deviations (SD) between tree species. All factors are scaled by subtracting the mean and dividing by standard deviation. All variables except tree replicates, sampling season, and tree richness are log + 1 transformed. Body weight values are also cube root transformed. Standardized parameter estimates (with standard errors, t, and *p*-values) are shown for explanatory variables. *p*-values in bold mean *p* ≤ 0.05, *p*-values of italic mean *p* ≤ 0.1.

	Caterpillar Species Richness SD			Abundance SD		
	*Est ± SE*	*t*	*p*	*Est ± SE*	*t*	*p*
(Intercept)	0.996 ± 0.089	11.141	**<0.001**	1.459 ± 0.097	15.026	**<0.001**
Tree replicates	0.006 ± 0.130	0.047	0.963	-	-	**-**
Caterpillar species richness	-	-	-	0.451 ± 0.115	3.937	**0.002**
Sampling season	−0.017 ± 0.093	−0.184	0.857	−0.117 ± 0.105	−1.110	0.291
Tree richness	−0.047 ± 0.130	−0.363	0.724	−0.004 ± 0.110	−0.036	0.972
	**Caterpillar FDis SD**			**Caterpillar generalism SD**		
	*Est ± SE*	*t*	*p*	*Est ± SE*	*t*	*p*
(Intercept)	0.662 ± 0.031	21.122	**<0.001**	1.41 ± 0.042	33.862	**<0.001**
Caterpillar species richness	−0.132 ± 0.037	−3.575	**0.004**	−0.078 ± 0.049	−1.580	0.142
Sampling season	0.019 ± 0.034	0.550	0.594	0.126 ± 0.045	2.772	**0.018**
Tree species richness	−0.075 ± 0.036	−2.115	*0.058*	0.030 ± 0.048	0.631	0.541
	**Caterpillar body weight SD**			**Caterpillar head capsule width SD**		
	*Est ± SE*	*t*	*p*	*Est ± SE*	*t*	*p*
(Intercept)	0.550 ± 0.042	13.063	**<0.001**	0.322 ± 0.020	15.998	**<0.001**
Caterpillar species richness	−0.129 ± 0.050	−2.587	**0.025**	−0.096 ± 0.024	−4.039	**0.002**
Sampling season	0.016 ± 0.046	0.351	0.733	−0.028 ± 0.022	−1.291	0.223
Tree species richness	−0.092 ± 0.048	−1.930	*0.080*	−0.067 ± 0.023	−2.934	**0.014**
	**Caterpillar relative head capsule width SD**					
	*Est ± SE*	*t*	*p*			
(Intercept)	0.096 ± 0.011	8.763	**<0.001**			
Caterpillar species richness	−0.016 ± 0.016	−1.007	0.337			
Caterpillar body weight	0.004 ± 0.015	0.254	0.805			
Sampling season	<0.001 ± 0.012	0.007	0.995			
Tree species richness	0.002 ± 0.014	0.169	0.869			
	**Caterpillar hair coverage SD**			**Caterpillar aposematism SD**		
	*Est ± SE*	*t*	*p*	*Est ± SE*	*t*	*p*
(Intercept)	0.615 ± 0.023	26.604	**<0.001**	0.192 ± 0.011	17.872	**<0.001**
Caterpillar species richness	−0.132 ± 0.027	−4.825	**0.001**	−0.066 ± 0.013	−5.177	**<0.001**
Sampling season	−0.006 ± 0.025	−0.221	0.829	0.067 ± 0.012	5.737	**<0.001**
Tree species richness	−0.057 ± 0.026	−2.161	*0.054*	−0.026 ± 0.012	−2.159	*0.054*

**Table A2:** Summary of linear model testing of changes in beta diversity estimates between tree species. Analysis run similarly for overall beta diversity and its two components. All factors are scaled by subtracting the mean and dividing by standard deviation. Response factors and number of sampled plots are log + 1 transformed. Standardized parameter estimates (with standard errors, t, and *p*-values) are shown for explanatory variables. *p*-values in bold mean *p* ≤ 0.05, *p*-values of italic mean *p* ≤ 0.1.

	Caterpillar Species Turnover			Caterpillar Species Nestedness		
	*Est ± SE*	*t*	*p*	*Est ± SE*	*t*	*p*
(Intercept)	0.627 ± 0.005	126.823	**<0.001**	0.043 ± 0.005	9.471	**<0.001**
Number of sampled plots	−0.012 ± 0.007	−1.769	0.105	0.007 ± 0.006	1.165	0.269
Sampling season	0.016 ± 0.005	3.183	**0.009**	−0.009 ± 0.005	−1.863	*0.089*
Tree species richness	−0.023 ± 0.007	−3.340	**0.007**	0.012 ± 0.006	1.827	*0.095*
	**Caterpillar beta diversity**					
	*Est ± SE*	*t*	*p*			
(Intercept)	0.651 ± 0.003	225.127	**<0.001**			
Number of sampled plots	−0.008 ± 0.004	−1.983	*0.073*			
Sampling season	0.011 ± 0.003	3.776	**0.003**			
Tree species richness	−0.016 ± 0.004	−4.029	**0.002**			
