# Supplementary material for: Leaf Nutritional Content, Tree Richness, and Season Shape the Caterpillar Functional Trait Composition Hosted by Trees
Source: Insects. 2022 Nov 29;13(12):1100. doi: 10.3390/insects13121100 (PMC9785672; doi:10.3390/insects13121100)
Supplement: Supplementary file 1 [file insects-13-01100-s001.zip › insects-2061622-supplementary.pdf]

Supplement 1

Leaf trait measurements and selection for analysis

Leaf samples were collected between August and October 2018, and comprised only fully developed, non-senescent leaves free of damage from herbivores, pathogens or mechanical stress. Leaves were sampled from all 16 tree species in all richness levels (monocultures, 2-, 4-, 8-, 16- and 24-species mixtures), including several tree replicates each and several leaf samples from each tree's crown. In total, this amounted to more than 2 000 leaf samples (see also, [54]). Because of this high number of samples, leaf traits were not measured directly, but 'predicted' via reflectance spectroscopy. For this purpose, a so called 'calibration set' was collected, composed of ten samples per species (10 to 25 leaves each) across all richness levels, randomly positioned within the crown of several randomly selected trees for each species.

For all leaves (predicted samples as well as calibration samples), reflectance spectra from fresh leaf samples with an ASD FieldSpec® 4 Wide Resolution Spectroradiometer (Malvern Panalytical Ltd., Malvern, United Kingdom) across 250 to 2500 nm was measured. The equipment was optimized regularly with a calibration panel (Spectralon®, Labsphere, Durham, New Hampshire, USA).
Only the calibration leaves were processed for direct trait measurements: saturated fresh leaves were weighed for each sample, scanned and weighed again after 72 hours drying at 80 °C. Image leaf area was analyzed (WinFOLIA software, Regent Instruments, Quebec, Canada) and specific leaf area (leaf area / leaf dry mass) as well as leaf dry matter content (leaf dry mass / leaf fresh mass) were calculated [62]. Dried calibration leaves were then ground to fine powder from which 200 mg were processed by a nitric acid digestion. Magnesium, calcium and potassium contents were analyzed from the filtrate via atomic absorption spectrometry (ContrAA 300 AAS, Analytik Jena, Jena, Germany), and phosphorus content with a spectrophotometric assay using an acid molybdate technique. For carbon and nitrogen content, 5 mg of leaf powder were analyzed with an elemental analyzer (Vario EL Cube, Elementar, Langenselbold, Germany).

Finally, spectra of the calibration samples were analyzed with the Unscrambler X software (version 10.1, CAMO Analytics, Oslo, Norway). Individual pre-treatments per leaf trait were applied to the spectral data to reveal relevant parts of the spectra and to reduce noise. For each trait, the pre-treated spectral data were used to fit a Partial Least Square regression model (PLS). In the last step, these PLS models were used to predict the true samples' trait values based on their reflectance spectra. The predicted trait values were averaged per species per tree richness level.

Table S1. Sampling details per tree species. Amount of sampled trees per species for each richness level per season, and the respective caterpillar species richness and abundance.

Tree species	Season	Tree richness	Tree replicates	Caterpillar richness	Caterpillar abundance
Castanea henryi	Autumn	1	5	4	8
Castanea henryi	Autumn	2	8	2	3
Castanea henryi	Autumn	4	6	3	4
Castanea henryi	Autumn	8	7	0	0
Castanea henryi	Autumn	16	5	3	3
Castanea henryi	Spring	1	6	4	4
Castanea henryi	Spring	2	8	0	0
Castanea henryi	Spring	4	6	0	0
Castanea henryi	Spring	8	4	1	1
Castanea henryi	Spring	16	5	1	1
Castanea henryi	Summer	1	5	7	10
Castanea henryi	Summer	2	9	5	6
Castanea henryi	Summer	4	6	3	5
Castanea henryi	Summer	8	5	6	8
Castanea henryi	Summer	16	5	10	16
Castanopsis eyrei	Autumn	1	4	1	1
Castanopsis eyrei	Autumn	2	1	1	1
Castanopsis eyrei	Autumn	4	1	0	0
Castanopsis eyrei	Autumn	8	4	3	4
Castanopsis eyrei	Autumn	16	2	0	0
Castanopsis eyrei	Spring	1	4	0	0
Castanopsis eyrei	Spring	2	1	1	1
Castanopsis eyrei	Spring	4	1	0	0
Castanopsis eyrei	Spring	8	4	4	4
Castanopsis eyrei	Spring	16	2	0	0
Castanopsis eyrei	Summer	1	4	3	3
Castanopsis eyrei	Summer	2	1	2	3
Castanopsis eyrei	Summer	4	1	1	1
Castanopsis eyrei	Summer	8	4	5	8
Castanopsis eyrei	Summer	16	2	7	15
Castanopsis sclerophylla	Autumn	1	8	5	5
Castanopsis sclerophylla	Autumn	2	9	1	1
Castanopsis sclerophylla	Autumn	4	5	2	2
Castanopsis sclerophylla	Autumn	8	5	1	1
Castanopsis sclerophylla	Autumn	16	5	0	0
Castanopsis sclerophylla	Spring	1	8	2	2
Castanopsis sclerophylla	Spring	2	9	3	3
Castanopsis sclerophylla	Spring	4	5	2	2
Castanopsis sclerophylla	Spring	8	5	2	2
Castanopsis sclerophylla	Spring	16	5	3	4

Castanopsis sclerophylla	Summer	1	8	6	12
Castanopsis sclerophylla	Summer	2	9	10	13
Castanopsis sclerophylla	Summer	4	5	10	12
Castanopsis sclerophylla	Summer	8	5	9	9
Castanopsis sclerophylla	Summer	16	5	4	5
Choerospondias axillaris	Autumn	1	6	1	1
Choerospondias axillaris	Autumn	2	10	4	5
Choerospondias axillaris	Autumn	4	5	3	10
Choerospondias axillaris	Autumn	8	5	0	0
Choerospondias axillaris	Autumn	16	5	1	2
Choerospondias axillaris	Spring	1	2	1	1
Choerospondias axillaris	Spring	2	9	3	3
Choerospondias axillaris	Spring	4	5	0	0
Choerospondias axillaris	Spring	8	2	0	0
Choerospondias axillaris	Spring	16	5	0	0
Choerospondias axillaris	Summer	1	6	1	1
Choerospondias axillaris	Summer	2	10	4	5
Choerospondias axillaris	Summer	4	4	1	3
Choerospondias axillaris	Summer	8	5	1	1
Choerospondias axillaris	Summer	16	5	4	5
Cyclobalanopsis myrsinifolia	Autumn	1	8	3	3
Cyclobalanopsis myrsinifolia	Autumn	2	9	3	4
Cyclobalanopsis myrsinifolia	Autumn	4	5	2	2
Cyclobalanopsis myrsinifolia	Autumn	8	8	0	0
Cyclobalanopsis myrsinifolia	Autumn	16	5	1	1
Cyclobalanopsis myrsinifolia	Spring	1	10	8	10
Cyclobalanopsis myrsinifolia	Spring	2	9	3	3
Cyclobalanopsis myrsinifolia	Spring	4	4	3	4
Cyclobalanopsis myrsinifolia	Spring	8	8	5	8
Cyclobalanopsis myrsinifolia	Spring	16	5	0	0
Cyclobalanopsis myrsinifolia	Summer	1	8	11	47
Cyclobalanopsis myrsinifolia	Summer	2	9	12	36
Cyclobalanopsis myrsinifolia	Summer	4	5	3	4
Cyclobalanopsis myrsinifolia	Summer	8	8	14	21
Cyclobalanopsis myrsinifolia	Summer	16	5	4	5
Koelreuteria bipinnata	Autumn	1	6	2	4
Koelreuteria bipinnata	Autumn	2	12	4	7
Koelreuteria bipinnata	Autumn	4	8	2	2
Koelreuteria bipinnata	Autumn	8	5	2	2
Koelreuteria bipinnata	Autumn	16	4	0	0
Koelreuteria bipinnata	Spring	1	6	0	0
Koelreuteria bipinnata	Spring	2	10	4	6
Koelreuteria bipinnata	Spring	4	8	1	1
Koelreuteria bipinnata	Spring	8	5	0	0

Koelreuteria bipinnata	Spring	16	4	0	0
Koelreuteria bipinnata	Summer	1	6	4	4
Koelreuteria bipinnata	Summer	2	13	12	29
Koelreuteria bipinnata	Summer	4	8	4	5
Koelreuteria bipinnata	Summer	8	5	2	2
Koelreuteria bipinnata	Summer	16	4	0	0
Liquidambar formosana	Autumn	1	6	2	2
Liquidambar formosana	Autumn	2	9	3	3
Liquidambar formosana	Autumn	4	5	2	2
Liquidambar formosana	Autumn	8	4	2	2
Liquidambar formosana	Autumn	16	6	5	5
Liquidambar formosana	Spring	1	6	2	2
Liquidambar formosana	Spring	2	9	1	2
Liquidambar formosana	Spring	4	5	1	10
Liquidambar formosana	Spring	8	4	3	3
Liquidambar formosana	Spring	16	6	0	0
Liquidambar formosana	Summer	1	6	5	9
Liquidambar formosana	Summer	2	9	6	11
Liquidambar formosana	Summer	4	5	3	7
Liquidambar formosana	Summer	8	4	5	8
Liquidambar formosana	Summer	16	7	8	12
Lithocarpus glaber	Autumn	1	6	0	0
Lithocarpus glaber	Autumn	2	10	3	3
Lithocarpus glaber	Autumn	4	10	2	2
Lithocarpus glaber	Autumn	8	5	1	1
Lithocarpus glaber	Autumn	16	5	3	3
Lithocarpus glaber	Spring	1	6	2	2
Lithocarpus glaber	Spring	2	10	2	2
Lithocarpus glaber	Spring	4	9	2	2
Lithocarpus glaber	Spring	8	5	1	1
Lithocarpus glaber	Spring	16	5	0	0
Lithocarpus glaber	Summer	1	6	5	6
Lithocarpus glaber	Summer	2	11	10	25
Lithocarpus glaber	Summer	4	10	12	29
Lithocarpus glaber	Summer	8	5	6	7
Lithocarpus glaber	Summer	16	5	8	10
Nyssa sinensis	Autumn	1	6	0	0
Nyssa sinensis	Autumn	2	10	0	0
Nyssa sinensis	Autumn	4	4	0	0
Nyssa sinensis	Autumn	8	4	0	0
Nyssa sinensis	Autumn	16	5	3	4
Nyssa sinensis	Spring	1	6	1	1
Nyssa sinensis	Spring	2	9	4	5
Nyssa sinensis	Spring	4	4	0	0

Nyssa sinensis	Spring	8	5	0	0
Nyssa sinensis	Spring	16	5	0	0
Nyssa sinensis	Summer	1	6	4	6
Nyssa sinensis	Summer	2	9	3	4
Nyssa sinensis	Summer	4	4	2	4
Nyssa sinensis	Summer	8	5	7	12
Nyssa sinensis	Summer	16	5	5	8
Quercus fabri	Autumn	1	6	3	5
Quercus fabri	Autumn	2	9	2	3
Quercus fabri	Autumn	4	4	2	3
Quercus fabri	Autumn	8	4	0	0
Quercus fabri	Autumn	16	3	0	0
Quercus fabri	Spring	1	6	1	1
Quercus fabri	Spring	2	9	2	2
Quercus fabri	Spring	4	5	3	3
Quercus fabri	Spring	8	4	1	1
Quercus fabri	Spring	16	3	1	1
Quercus fabri	Summer	1	6	3	4
Quercus fabri	Summer	2	9	4	5
Quercus fabri	Summer	4	5	7	22
Quercus fabri	Summer	8	4	5	5
Quercus fabri	Summer	16	3	6	9
Quercus glauca	Autumn	1	8	3	5
Quercus glauca	Autumn	2	9	7	11
Quercus glauca	Autumn	4	5	3	4
Quercus glauca	Autumn	8	5	3	7
Quercus glauca	Autumn	16	4	0	0
Quercus glauca	Spring	1	6	0	0
Quercus glauca	Spring	2	9	5	5
Quercus glauca	Spring	4	5	3	3
Quercus glauca	Spring	8	5	2	2
Quercus glauca	Spring	16	4	3	3
Quercus glauca	Summer	1	8	7	15
Quercus glauca	Summer	2	9	9	19
Quercus glauca	Summer	4	5	5	9
Quercus glauca	Summer	8	5	5	9
Quercus glauca	Summer	16	3	4	5
Quercus serrata	Autumn	1	6	3	3
Quercus serrata	Autumn	2	9	1	1
Quercus serrata	Autumn	4	5	4	4
Quercus serrata	Autumn	8	5	1	1
Quercus serrata	Autumn	16	4	2	2
Quercus serrata	Spring	1	6	3	3
Quercus serrata	Spring	2	9	4	5

Quercus serrata	Spring	4	5	0	0
Quercus serrata	Spring	8	5	2	2
Quercus serrata	Spring	16	5	3	3
Quercus serrata	Summer	1	6	3	4
Quercus serrata	Summer	2	9	5	7
Quercus serrata	Summer	4	5	3	8
Quercus serrata	Summer	8	5	4	4
Quercus serrata	Summer	16	5	8	12
Rhus chinensis	Autumn	1	4	0	0
Rhus chinensis	Autumn	4	3	1	1
Rhus chinensis	Autumn	8	2	1	1
Rhus chinensis	Spring	1	4	0	0
Rhus chinensis	Spring	4	4	0	0
Rhus chinensis	Spring	8	2	0	0
Rhus chinensis	Spring	16	1	0	0
Rhus chinensis	Summer	1	4	1	1
Rhus chinensis	Summer	4	4	3	6
Rhus chinensis	Summer	8	2	1	2
Rhus chinensis	Summer	16	1	3	5
Sapindus mukorossi	Autumn	1	6	1	1
Triadica sebifera	Autumn	1	6	0	0
Triadica sebifera	Autumn	2	9	1	1
Triadica sebifera	Autumn	4	5	4	4
Triadica sebifera	Autumn	8	3	2	2
Triadica sebifera	Autumn	16	5	1	1
Triadica sebifera	Spring	1	6	2	2
Triadica sebifera	Spring	2	7	5	6
Triadica sebifera	Spring	4	5	1	1
Triadica sebifera	Spring	8	2	0	0
Triadica sebifera	Spring	16	5	0	0
Triadica sebifera	Summer	1	6	5	7
Triadica sebifera	Summer	2	8	5	5
Triadica sebifera	Summer	4	5	1	1
Triadica sebifera	Summer	8	2	1	1
Triadica sebifera	Summer	16	5	5	8
Sapindus mukorossi	Autumn	2	9	3	3
Sapindus mukorossi	Autumn	4	5	1	1
Sapindus mukorossi	Autumn	8	8	0	0
Sapindus mukorossi	Autumn	16	6	1	2
Sapindus mukorossi	Spring	1	6	1	1
Sapindus mukorossi	Spring	2	9	3	4
Sapindus mukorossi	Spring	4	5	0	0
Sapindus mukorossi	Spring	8	5	1	1
Sapindus mukorossi	Spring	16	6	3	4

Sapindus mukorossi	Summer	1	6	3	4
Sapindus mukorossi	Summer	2	9	6	6
Sapindus mukorossi	Summer	4	5	3	5
Sapindus mukorossi	Summer	8	5	3	3
Sapindus mukorossi	Summer	16	6	2	2
Schima superba	Autumn	1	16	1	1
Schima superba	Autumn	4	7	3	5
Schima superba	Autumn	8	6	2	2
Schima superba	Autumn	16	4	0	0
Schima superba	Spring	1	16	3	3
Schima superba	Spring	4	7	1	1
Schima superba	Spring	8	6	3	3
Schima superba	Spring	16	4	0	0
Schima superba	Summer	1	16	6	13
Schima superba	Summer	4	7	5	12
Schima superba	Summer	8	6	11	15
Schima superba	Summer	16	4	6	8

Table S2. Lepidoptera samples per family. Lepidoptera species composition and abundance at the rank of family, across all tree species, richness levels, and seasons.

Family	Richness	Abundance
Erebidae	48	309
Geometridae	33	573
Notodontidae	11	22
Tortricidae	7	9
Noctuidae	6	7
Nolidae	3	4
Sphingidae	2	33
Pyralidae	2	10
Psychidae	2	4
Drepanidae	2	2
Gelechiidae	2	2
Limacodidae	2	2
Lasiocampidae	1	10
Unassigned Lepidoptera	25	33

Table S3. Caterpillar generalism. Number of caterpillar species per each potential generalism class, i.e. how many caterpillar species were found from certain amount of tree species.

Generalism	1	2	3	4	5	6	7	8	9	10	11	12	13	14	15	16
Number of caterpillar species	92	20	5	2	5	4	4	2	3	1	1	1	0	2	2	1

Table S4. Sensitivity analysis. Summary results of linear mixed-effects models after fixed factor reduction using backward selection with criterion P < 0.05 for the averaged caterpillar traits per tree species per richness level per season, after removing 11 most common species that were found from over half of the tree species in the study. All factors are scaled by subtracting the mean and dividing by standard deviation. Body weight values are also cube root transformed. All variables except number of tree replicates, sampling season and tree richness are log + 1 transformed. Standardized parameter estimates (with standard errors, t and p values) are shown for the variables retained in the minimal models. P-values in bold mean P ≤ 0.05.

	**Caterpillar species richness**			**Caterpillar abundance**		
	Est±SE	*t*	*P*	*Est±SE*	*t*	*P*
(Intercept)	1.051± 0.035	29.765	**<0.001**	1.158± 0.023	49.701	**<0.001**
Tree replicates	0.079± 0.030	2.611	**0.010**	-	-	-
Caterpillar species richness	-	-	-	0.411±0.018	23.324	**<0.001**
Sampling **s**eason	-	-	-	0.015±0.017	0.850	0.397
Leaf C content	-	-	-	-0.009±0.022	-0.413	0.684
Season: leaf C content	-	-	-	0.067± 0.018	3.795	**<0.001**
	**Caterpillar FDis**			**Caterpillar generalism**		
	Est±SE	*t*	*P*	*Est±SE*	*t*	*P*
(Intercept)	0.543±0.025	21.320	**<0.001**	1.458±0.032	44.994	**<0.001**
Caterpillar species richness	0.381±0.025	15.020	**<0.001**	-	-	**-**
Caterpillar body weight	-	-	**-**	-0.100±0.034	-2.967	**0.003**
Sampling **s**eason	-	-	-	-0.200±0.034	-5.909	**<0.001**
Tree species richness	-	-	-	0.017±0.033	0.526	0.600
Leaf Mg content	-	-	-	0.012±0.033	0.361	0.718
Tree richness: Mg	-	-	-	-0.102±0.033	-3.075	**0.002**
	**Caterpillar body weight**			**Caterpillar head capsule width**		
	Est±SE	*t*	*P*	*Est±SE*	*t*	*P*
(Intercept)	1.052±0.029	36.015	**<0.001**	0.711±0.008	86.239	**<0.001**
Caterpillar body weight	-	-	-	0.253±0.008	30.554	**<0.001**
Sampling **s**eason	-0.097±0.029	-3.319	**0.001**	-	-	-
Leaf Mg content	-	-	-	-0.022±0.008	-2.641	**0.009**
	**Caterpillar relative head capsule width**					
	Est±SE	*t*	*P*			
(Intercept)	0.455±0.006	72.178	**<0.001**			
Caterpillar body weight	-0.035±0.006	-5.426	**<0.001**			
Tree richness	-0.001±0.006	-0.177	0.860			

Leaf C content	-0.017±0.008	-2.207	**0.029**			
Leaf Mg content	-0.019±0.008	-2.398	**0.018**			
Tree richness: Mg	-0.014±0.006	-2.103	**0.037**			
	**Caterpillar hair coverage**			**Caterpillar aposematism**		
	Est±SE	*t*	*P*	*Est±SE*	*t*	*P*
(Intercept)	0.656±0.040	16.332	**<0.001**	0.171±0.017	9.897	**<0.001**
Caterpillar body weight	-	-	**-**	0.059±0.018	3.320	**0.001**
Aposematism	0.191±0.042	4.509	**<0.001**	-	-	-
Hair coverage	-	-	-	0.075±0.017	4.323	**<0.001**
Sampling **s**eason	-0.144±0.041	-3.489	**<0.001**	0.074±0.018	4.085	**<0.001**
Leaf C content	-0.098±0.046	-2.142	**0.034**	0.058±0.017	3.343	**0.004**
Leaf N content	-0.137±0.045	-3.062	**0.003**	-	-	-
Season: C	-	-	-	0.035±0.017	2.032	**0.044**

Table S5. Caterpillar trait intra- versus interspecific variation. Intraspecific variation was estimated as the average of standard deviations (SD) of each species that had more than one individual. The interspecific variation was estimated as standard deviation between caterpillar trait averages of each caterpillar species across all sampling units.

Trait variation (SD)	***Body weight (mg CBRT)***	***Head capsule width (mm)***	***Relative head capsule width***	***Hair coverage***	***Aposematism***
Intraspecific	0.74	0.36	0.14	0	0
Interspecific	1.71	0.78	0.15	1.33	0.42

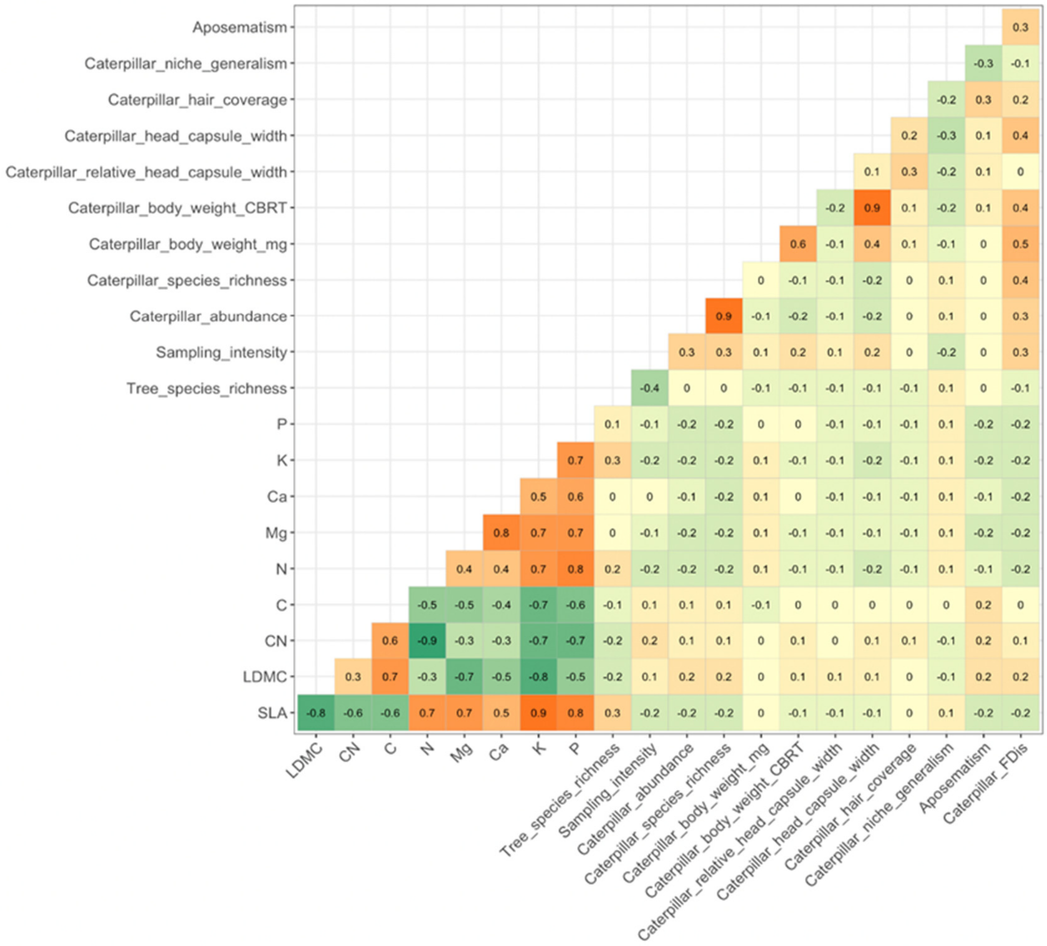

Figure S1. Variable correlations. Relationships among all potential and used response- and predictor variables for the analyses. Values are Pearson's correlation coefficients r. Leaf trait values are averaged per tree species per tree richness level, and caterpillar traits per tree species per tree richness level per season.

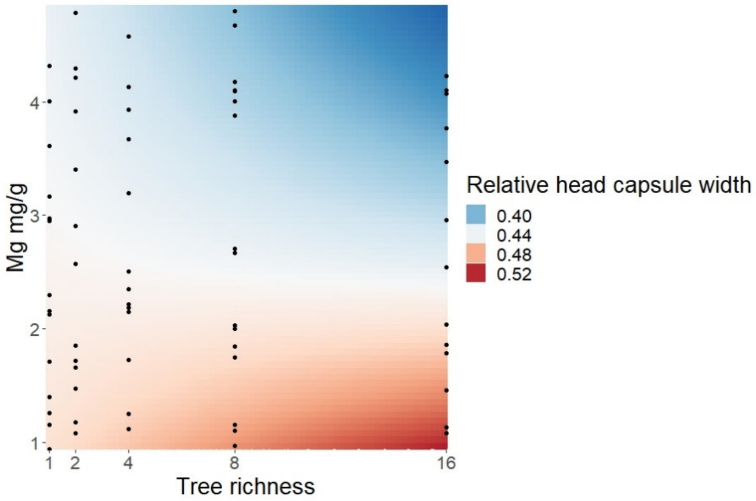

Figure S2. Relative head capsule width sensitivity analysis. Relationship of relative head capsule width to interaction of Mg (mg/g) content and tree richness. Color gradient represents estimated change in response values, and point clouds show observed values. Relative head capsule width and Mg content values are log + 1 transformed.
